# Exploring food insecurity and multimorbidity in Indian socially disadvantaged people: cross-sectional findings from LASI, 2017–18

**DOI:** 10.1186/s12889-023-16132-6

**Published:** 2023-06-26

**Authors:** Salmaan Ansari, Abhishek Anand, Shalini Singh, Babul Hossain

**Affiliations:** 1 International Institute for Population Sciences, Mumbai, 400088 India; 2 Department of Demography, University of Vienna, Vienna, Austria; 3 Centre for Health Services Studies, University of Kent, Canterbury, United Kingdom

**Keywords:** Multimorbidity, Food insecurity, Aging, India

## Abstract

**Objectives:**

The present study aimed to examine the association of multimorbidity status with food insecurity among disadvantaged groups such as Scheduled Castes (SCs), Scheduled Tribes (STs), and Other Backward Class (OBCs) in India.

**Method:**

The data for this study was derived from the first wave of the Longitudinal Ageing Study in India (LASI),2017–18, focusing on 46,953 individuals aged 45 years and over who belong to SCs, STs, and OBCs groups. Food insecurity was measured based on the set of five questions developed by the Food and Nutrition Technical Assistance Program (FANTA). Bivariate analysis was performed to examine the prevalence of food insecurity by multimorbidity status along with socio-demographic and health-related factors. Multivariable logistic regression analysis and interaction models were used.

**Results:**

The overall prevalence of multimorbidity was about 16% of the study sample. The prevalence of food insecurity was higher among people with multimorbidity compared to those without multimorbidity. Unadjusted and adjusted models suggested that people with multimorbidity were more likely to be food insecure than people without multimorbidity. While middle-aged adults with multimorbidity and men with multimorbidity had a higher risk of food insecurity.

**Conclusion:**

The findings of this study suggest an association between multimorbidity and food insecurity among socially disadvantaged people in India. Middle-aged adults experiencing food insecurity tend to reduce the quality of their diet and consume a few low-cost, nutritionally deficient meals to maintain caloric intake, putting them again at risk for several negative health outcomes. Therefore, strengthening disease management could reduce food insecurity in those facing multimorbidity.

## Introduction

With economic progress and demographic change, India is going through an epidemiological shift leading to a dual burden of diseases, with communicable diseases becoming an additional burden as non-communicable diseases (NCDs) become more prevalent [1]. However, population ageing has contributed to an increasing trend NCDs, which are highly common among the elderly, putting them at risk of multiple chronic diseases as they age [2].

The occurrence of multiple health problems (i.e., ≥ 2 chronic conditions) is known as ‘Multimorbidity’ [3]. Multimorbidity, the co-occurrence of two or more chronic medical conditions within an individual, increase with age and is highly prevalent among those patients attending primary healthcare settings [4–6]. Multimorbidity in the older population has long been identified as a key barrier to living a healthy life, putting them at risk of negative health outcomes such as declining physical functioning, low quality of life, poor self-rated health, poor mental health, and mortality [7–10]. Furthermore, multiple morbidities and co-morbid conditions present a significant barrier to healthcare choice and disease management, leading to the development of poor health outcomes and, as a result, a rise in financial burden due to greater medical costs [11, 12]. Also, the existence of multiple chronic conditions (MCC) results in out-of-pocket health care expenditures that exert budget pressure on low-income households that further contributes to the economic vulnerability of older people which may increase the risk of food insecurity [13, 14].

Food insecurity can be defined as ‘when all people, at all times, do not have physical and economic access to sufficient, safe and nutritious food to meet their dietary needs and food preferences for active and healthy life [15]. Food insecurity is a problem for people across their lifespan, and it is a growing issue among older people resulting in socioeconomic deprivation due to increasing medical costs [11, 16]. Multiple chronic illnesses impair physical functions and create a barrier to generating economic resources, both of which have a direct influence on the quality of life and social concerns such as economic dependence or support systems of the older population [12, 17, 18]. Therefore, food insecurity is particularly a concern for older adults who suffer from multiple chronic conditions due to their vulnerability in experiencing several physical, psychological, financial, and social obstacles related to food access. Globally, food insecurity has emerged as one of the key challenges affecting millions of people, particularly older adults [19].

Several studies have looked at the health effects of food insecurity, and the findings support the hypothesis that food insecurity impacts nutritional status and dietary consumption, which is linked to poor health outcomes and low well-being in later life [20]. However, a substantial body of evidence suggests that there may be a reverse association: the health condition of older individuals may be a driver of food insecurity [21–24]. A few studies have connected multimorbidity to food insecurity in developed countries. For example, according to an analysis of the Canadian Community Health Survey based on adults aged 18–64 years, respondents with multiple chronic conditions have a higher likelihood of food insecurity than those without chronic illnesses [25]. In another nationally representative study in the United States, the odds ratios for being food insecure were shown to be greater among older adults aged 50 or more years who had two or more chronic diseases compared to those who had a single or no morbidity [13]. Meanwhile, multiple studies have demonstrated that the added economic vulnerability of food insecurity can lead to trade-offs with chronic disease management, including purchasing food versus medication, greater subsequent health care needs, sub-optimal chronic disease management, and worse overall health [25–29]. However, there is limited evidence from developing countries like India that highlights multimorbidity as a significant risk factor that increases the risk of food insecurity.

Females have a higher life expectancy and poorer health outcomes than their older male counterparts, resulting in gender differences in the prevalence of multimorbidity [30]. To ensure food security at the household level, females play an important role; however, there are gender disparities in the likelihood of food insecurity at the individual level [31]. Health-related gender disparities have been observed across the lifespan, while it becomes more prevalent in the ageing population due to differences in social and personal resources [32]. Old age, in particular, is a stage of life marked by considerable changes in societal norms, gender-related expectations, and family status. Due to disparities in social and economic aspects, it is necessary to look at the gender perspective in developing nations, especially in the Indian context. Therefore, we will also address the gender variations in the present study.

While looking at the social structure in India, Scheduled Castes (SCs), Scheduled Tribes (STs), and Other Backward Classes (OBCs) are the less privileged groups that often fare worse than the other groups across the social and economic indicators in India [33]. Individuals from these groups face social impairment and extreme poverty [34]. The individuals from SCs, STs, and OBCs groups lack purchasing power, live in substandard housing, and limited access to resources and entitlements [35]. In rural India, these marginalized populations are casual laborers performing a variety of available jobs. At the same time, in urban areas, they are urban poor employed as wage laborers at a variety of work sites, beggars, vendors, small service providers, and domestic help, among others, who live in slums and other makeshift shelters without access to social security [36]. Members of these communities are subjected to systematic violence, including denial of access to land, adequate housing, education, and jobs [35, 37]. The SCs, STs, and OBCs became eligible for some rights as Indian citizens, such as economic rights guaranteed by the Indian Constitution, people from these groups continue to perform poor in range of health indicators, starting with poor nutrition status, vaccination rates among children, and access to health care use across the age groups [38–40]. As a result, the poor social and economic aspect further susceptible these groups of individual to develop different morbidity and physical ailments which can further affect their basic need including the food and nutrition. These aspects thus suggested poorer physical health conditions and severe food insecurity among these disadvantaged groups.

**Fig. 1:**
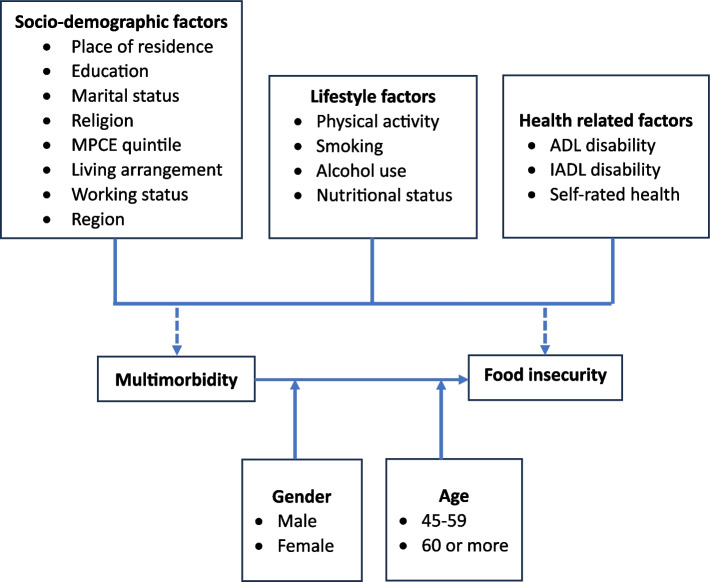
Preliminary theoretical model of the multimorbidity-food insecurity status association and its potential moderators

With this background, the present study is aimed at exploring the association of multimorbidity with food insecurity among the population of socially disadvantaged groups in India. We hypothesized that multimorbidity is associated with higher odds of food insecurity after adjusting for several sociodemographic and health-related confounders. Additionally, age and gender differences in the possible association between multimorbidity and food insecurity has been explored. Figure 1 shows a preliminary theoretical framework for this association, which illustrates that multimorbidity might directly influence food insecurity status of an Individual, though gender and age might change the strength or direction of the association.

## Material & methods

### Data source

The data for this study was derived from the first wave of the Longitudinal Ageing Study in India (LASI), a large-scale survey conducted during the year 2017–18. The LASI is the first survey of its kind in India, focusing on in-depth knowledge about the ageing population, addressing social, mental, and functional health, as well as their social and economic wellbeing, using an internationally comparable research design. This is a nationally representative survey that targeted people aged 45 and above (including spouses, irrespective of age), which included a panel sample of 72,2500 individuals from across all the states (excluding Sikkim) and union territories of the country. As a longitudinal database, it aims to follow a representative sample every two years for the next 25 years with refreshment samples for attrition due to death, dislocation, non-contact, and refusal. The LASI is a collaborative study between the International Institute for Population Sciences (IIPS), Harvard T. H. Chan School of Public Health (HSPH), and the University of Southern California (USC). The first wave of this survey received financial support from the Ministry of Health and Family Welfare (MoHFW), Govt. of India, the National Institute on Aging (NIA/NIH), the USA, and the United Nations Population Fund (UNFP), India.

The LASI wave 1 adopted a multistage stratified area probability cluster sampling design, including a three-stage sampling design in rural areas and a four-stage sampling design in urban areas. In each state/UT, the first stage involved selecting Primary Sampling Units (PSUs), that is, sub-districts (Tehsils/Talukas), and the second stage involved the selection of villages in rural areas and wards in urban areas in the selected PSUs. In rural areas, households were selected from selected villages in the third stage. However, sampling in urban areas involved an additional stage. Specifically, in the third stage, one Census Enumeration Block (CEB) was randomly selected in each urban area. In the fourth stage, households were selected from this CEB. The survey report included a detailed methodology section with all the information about the survey design and data collection [40].

### Study population

The present study was restricted to eligible respondents aged 45 years and above. Furthermore, the study considered individuals belonging to SC/ST/OBC categories in a broad age group of 45 to 59 years and 60 years and above. The final analytical sample included 46,953 older persons aged 45 years and over.

## Measures

The outcome variable for this study is food insecurity. The LASI used a set of five questions developed by the Food and Nutrition Technical Assistance Program (FANTA) to collect data on food availability [41]. The main explanatory variable for this study is multimorbidity of chronic diseases and was defined as an accumulation of two or more chronic diseases [10, 42]. Several potential covariates were selected from the survey including lifestyle factors, health-related factors and socio-demographic factors. The variables used for this study from the LASI survey are described in Tables 1 and 2.

**Table 1:** Variable description

Variable name	Question	Response options	Measured variable
**Outcome variable**			
** Food insecurity**	Over the years, **(1)** did you ever reduce the size of your meal? **(2)** did you eat enough food of your choice? **(3)** were you hungry but didn’t eat because there was not enough food in your household? **(4)** did you ever not eat for a whole day because there was not enough food in your household? and **(5)** do you think you have lost weight in the last 12 months because there was not enough food in your household?	1 = Yes, 2 = No	Participants who responded affirmatively to one or more questions were classified as **1 “food insecure”**; otherwise, they were classified as **0 “food secure”** [42] **Note:** *For affirmative response, we reversed the code of second question*
**Main explanatory variable**			
** Multimorbidity**	Has any health professional ever diagnosed you with the following chronic conditions or diseases? 1. Hypertension 2. Diabetes mellitus 3. Cancer or malignant tumor 4. Chronic lung diseases 5. Chronic heart disease 6. Stroke 7. Bone-related diseases 8. Neurological/psychiatric diseases 9. High cholesterol	1 = Yes, 2 = No	Participants with two or more diseases were classified as **1 “Multimorbidity”** and with no or single chronic disease as **0 “No multimorbidity”**

**Table 2:** Variable description

Covariates	Categories
**Gender**	1 = Men 2 = Women
**Age-group (in years)**	1 = 45–59 (Middle-aged) 2 = 60 or more (Older aged)
**Place of residence**	1 = Urban 2 = Rural
**Educational attainment**	1 = No education 2 = Less than five years 3 = 5–10 years completed, 4 = 10 or more years of schooling
**Marital status**	1 = Not currently in marital union 2 = Currently in marital union
**Religion**	1 = Hindu 2 = Muslim 3 = Others
**Monthly per capita expenditure (MPCE)**	1 = Poor (representing poorest and poor) 2 = Middle 3 = Rich (representing richest and rich)
**Physical activity**	1 = Frequent 2 = Rare 3 = Never
**Currently smoking**	0 = No (Never/Not currently smoking 1 = Yes (Currently smoking)
**Alcohol use**	0 = No 1 = Yes
**Nutritional status**	1 = Underweight (BMI ≤ 18.4 kg/m^2^) 2 = Normal (BMI 18.5 to 24.9 kg/m^2^) 3 = Overweight (BMI 25 to 29.9 kg/m^2^) 4 = Obese (BMI ≥ 30 kg/m^2^)
**Activities of daily living (ADL)**	0 = No (No limitation in ADL) 1 = Yes (One or more limitation in ADL)
**Instrumental activities of daily living (IADL)**	0 = No (No limitation in IADL) 1 = Yes (One or more limitation in IADL)
**Regional geography**	1 = North 2 = Central 3 = East 4 = Northeast 5 = West 6 = South

## Statistical analysis

Descriptive analysis was performed to describe the study sample. For each study variable, unweighted frequency and weighted percentages were calculated. Bivariate analysis was performed to examine the prevalence of food insecurity by multimorbidity status along with socio-demographic and health-related factors. A Chi-square test was performed to check for intergroup differences in the prevalence of multimorbidity, as well as in the prevalence of food insecurity. Binary logistic regression analysis was performed to assess the adjusted association between food insecurity and each set of independent variables (socio-demographic and health-related factors). Further, a five-model multivariable logistics regression model with food insecurity as the dependent variable and multimorbidity as the main independent variable was performed to test the study hypothesis. In the first model, only multimorbidity as a main independent variable was entered. In the second model, sociodemographic factors were adjusted, whereas, in the third model, health-related factors were adjusted along with sociodemographic factors. As previously stated, gender and age are two significant independent variables in the study of the socially disadvantaged older population. Intersectional identity may aggravate several socioeconomic constraints, including morbidity patterns and food poverty. Hence, in the fourth model, gender differences in the association between morbidity status and food insecurity were examined by adding gender to the morbidity status interaction term (gender*multimorbidity). The fifth model included another interaction of multimorbidity with age (age*multimorbidity) to examine the interactive effect of age in the association between multimorbidity and food insecurity in socially disadvantaged people. Moreover, both the models with interaction terms were adjusted for sociodemographic and health-related factors. The sample weighting was taken into account to generate nationally representative estimates, therefore, the national sampling weights provided in LASI report were used in the analysis. We used the exponentiated regression coefficient – odds ratios (ORs) – as a measure of association. Also, 95% confidence intervals (95% CI) are reported. All the analysis was performed using STATA version 15, and the level of statistical significance was set at *P* < 0.05 [43].

## Results

### Characteristics of the participants

Table 3 presents the characteristics of the participants in the study sample. Of the total of 46,953 socially disadvantaged people, women accounted for nearly 46% and men for 54% approximately of the sample. Half of the participants in the study were in the medium age range (45–59 years), while the other half were elderly (60 years or more). The study sample consisted of about 72% people belonging to rural areas and around 28% from urban areas. In the sample, 56% of the people had no education, whereas nearly 14% had 10 or more years of schooling. About 78% of the people were currently in the marital union and 49% were currently working. Most of the study population belongs to the Hindu religion (84.5%), followed by Muslims (only 8.7%). Among the sample population, nearly 16% had ADL disability and around 38% had IADL disability. About 30% of the study participants lived in the southern region of the country, followed by the eastern (21.44%) and central region (21.33%).

**Table 3:** Socio-economic and health profile of socially disadvantaged people, 2017–18

*Variables*	*Category*	*N*	*%*
**Age (in years)**	45–59	24,754	50.42
	60 or more	22,199	49.58
**Gender**	Male	21,826	45.91
	Female	25,127	54.09
**Residence**	Rural	32,623	72.18
	Urban	14,321	27.82
**Educational attainment**	No Education	24,674	55.93
	less than 5 years	5,721	10.96
	5–9 years completed	10,042	18.87
	10 or more years of schooling	6,515	14.24
**Marital Status**	Currently in marital union	35,061	73.66
	Not in marital union	11,892	26.34
**Living alone**	No	45,177	96.09
	Yes	1,776	3.91
**Working status**	No	23,707	51.13
	Yes	23,237	48.87
**Religion**	Hindu	34,844	84.49
	Muslim	4,527	8.68
	Others	7,581	6.83
**MPCE Quintile**	Poor	20,795	45.26
	Middle	9,485	20.41
	Rich	16,673	34.34
**ADL disability**	No	40,503	83.86
	Yes	6,450	16.14
**IADL disability**	No	31,134	61.57
	Yes	15,819	38.43
**SRH**	Good	19,130	37.38
	Poor	27,188	62.62
**Current smoker**	No	39,944	86.39
	Yes	6,621	13.61
**Alcohol use**	No	40,809	89.65
	Yes	5,774	10.35
**Physical activity**	Frequent	5,533	57.55
	Ever	4,012	12.57
	Never	21,919	29.88
**Nutritional Status**	Normal weight	22,900	23.52
	Underweight	8,850	51.82
	Overweight/obese	10,840	24.65
**Regional geography**	North	6,280	9.98
	Central	7,046	21.33
	East	7,809	21.44
	North East	6,983	3.35
	West	5,667	14.47
	South	13,168	29.42
**Total**		**46,953**	**100**

*%* Percentage, *N* Frequency, *MPCE* Monthly per capita expenditure, *ADL* Activities of daily living, *IADL* Instrumental activities of daily living, *SRH* Self-Rated Health, Samples (N) are unweighted and % are weighted

### Association between explanatory variables and multimorbidity

Table 4 illustrates the bivariate and logistic regression estimates for multimorbidity among socially disadvantaged people in India. In the bivariate estimates, all factors were found to be statistically significantly associated with multimorbidity. Older adults aged 60 or more years had significantly higher odds of multimorbidity [aOR: 1.72; CI: 1.48–2.01] in comparison to those in age group 45–59 years. Men had higher odds of multimorbidity than women [aOR: 1.25, CI: 1.05–1.49]. People with higher education of 10 or more years had higher odds of multimorbidity [aOR: 1.51; CI: 1.14–2.01] than people with no education. People who were not currently in union had higher odds of multimorbidity than people who were currently in union [aOR: 1.05; CI: 0.89–1.24]. Muslim individuals had higher odds of multimorbidity than Hindu individuals [aOR: 1.25; CI: 1.04–1.50]. Respondent with ADL disability [aOR: 1.45; CI: 1.24–1.69], or with IADL disability [aOR:1.42; CI: 1.24–1.65] had higher odds of multimorbidity. People with poor SRH [aOR: 3.01; CI: 2.53–3.58], were currently smoking [aOR: 1.21; CI: 1.01–1.45], and consumed alcohol [aOR: 1.27; CI: 1.06–1.53] had higher odds of multimorbidity. Adults who were underweight had lower odds of multimorbidity than the people who had normal weight [aOR: 0.55; CI: 0.47–0.65].

**Table 4:** Bivariate and logistic regression estimates for multimorbidity among socially disadvantaged people, 2017–18

*Variables*	*Category*	*%*	*p-value*	*aOR (95% CI)*
**Age (in years)**	45–59	21.62	< 0.001	Ref
	60 or more	13.04		1.72*** (1.48,2.01)
**Gender**	Women	18.17	< 0.001	Ref
	Men	16.27		1.25** (1.05,1.49)
**Residence**	Rural	26.5	< 0.001	Ref
	Urban	13.75		1.46*** (1.24,1.72)
**Educational attainment**	No Education	14.74	< 0.001	Ref
	less than 5 years	17.82		1.25** (1.06,1.47)
	5–9 years	19.28		1.26* (1.03,1.51)
	10 or more years	24.29		1.51** (1.14,2.01)
**Marital Status**	Currently in marital union	16.15	< 0.001	Ref
	Not in marital union	20.5		1.05 (0.89,1.24)
**Living alone**	No	17.17	< 0.001	Ref
	Yes	20.39		1.03 (0.76,1.4)
**Currently working**	Yes	10.52	< 0.001	Ref
	No	23.78		1.79*** (1.56,2.07)
**Religion**	Hindu	16.41	< 0.001	Ref
	Muslim	24.79		1.25** (1.04,1.50)
	Others	18.65		0.94 (0.75,1.18)
**MPCE Quintile**	Poor	12.95	< 0.001	Ref
	Middle	16.8		1.24** (1.05,1.46)
	Rich	23.44		1.74*** (1.49,2.02)
**ADL disability**	No	14.91	< 0.001	Ref
	Yes	29.71		1.45*** (1.24,1.69)
**IADL disability**	No	13.37	< 0.001	Ref
	Yes	23.58		1.42*** (1.24,1.65)
**SRH**	Good	7.85	< 0.001	Ref
	Poor	22.83		3.01*** (2.53,3.58)
**Current smoker**	No	11.31	< 0.001	Ref
	Yes	18.3		1.21* (1.01,1.45)
**Alcohol use**	No	18	< 0.001	Ref
	Yes	11.58		1.27** (1.06,1.53)
**Physical activity**	Frequent	15.76	< 0.001	Ref
	Ever	16.66		1.12 (0.96,1.33)
	Never	20.74		1.13** (0.97,1.29)
**Nutritional Status**	Normal weight	14.64	< 0.001	Ref
	Underweight	9.22		0.55*** (0.47,0.65)
	Overweight/obese	28.02		1.87*** (1.55,2.26)
**Regional geography**	Central	9.2	< 0.001	Ref
	North	15.82		1.66*** (1.39,1.98)
	East	14.76		1.77*** (1.50,2.10)
	North East	9.91		1.12 (0.9,1.38)
	West	18.93		1.86*** (1.56,2.23)
	South	25.54		2.02*** (1.68,2.45)
**Total**		17.29		
**Pseduo R2**				**0.1542**

*%* Percentage, *aOR* adjusted Odds Ratio, *CI* Confidence interval, *MPCE* Monthly per capita expenditure, *ADL* Activities of daily living, *IADL* Instrumental activities of daily living, *SRH* Self-Rated Health

^*^ *p* < 0.05

^**^p < 0.005

^***^p < 0.001

### Association between multimorbidity and food insecurity

**Fig. 2:**
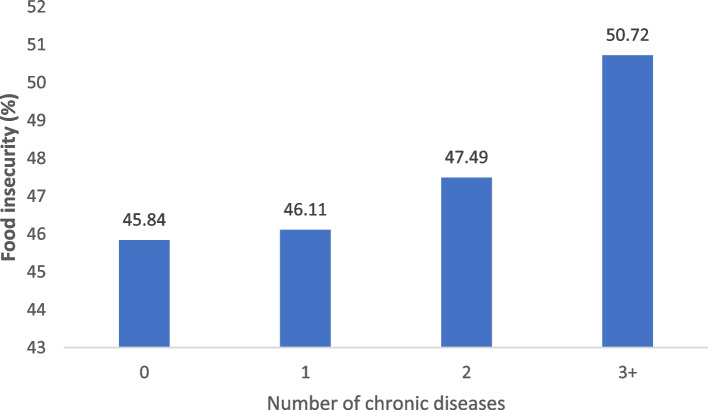
Prevalence of food insecurity by number of chronic diseases among socially disadvantaged people

**Fig. 3:**
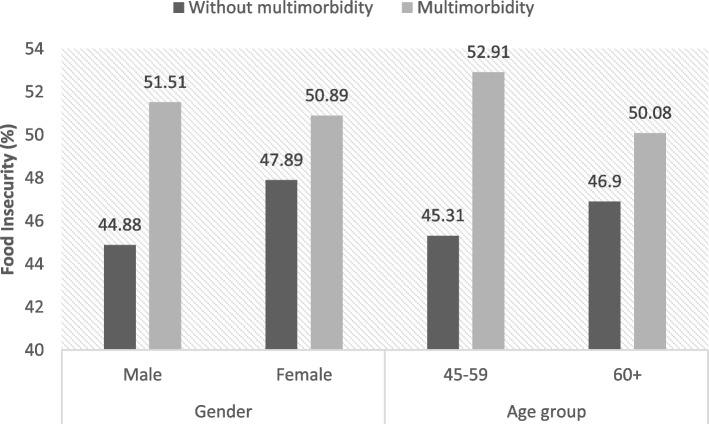
Prevalence of food insecurity by multimorbidity status among socially disadvantaged people

Figure 2 shows the prevalence of food insecurity by the number of chronic diseases. A linear association between the presence of chronic diseases and food insecurity can be observed with the prevalence of food insecurity rising from 45.84% in those with no chronic disease to 50.72% among those with three or more diseases. Figure 3 presents the prevalence of food insecurity with multimorbidity status by gender and age. It can be observed that food insecurity was found to be slightly higher in men with multimorbidity (51.51%) than in women with multimorbidity (50.51 percent). Meanwhile, the prevalence of food insecurity was higher among people with multimorbidity aged 45–59 years (52.91%) than older people with multimorbidity aged 60 or more years (50.08%).

Table 5 presents the bivariate estimates of food insecurity by multimorbidity status and other variables included in the study. It can be observed that food insecurity was found to be more prevalent among individuals with multimorbidity (51.16%) than among those without (46.06%).

**Table 5:** Bivariate estimates of food insecurity among socially disadvantaged people, 2017–18

*Variables*	*Category*	*%*	*p-value*
**Multimorbidity**	No	46.06	< 0.001
	Yes	51.16	
**Age-group (in years)**	45–59 Years	46.33	0.021
	60 + (Older aged)	47.32	
**Gender**	Women	47.76	0.068
	Men	45.97	
**Residence**	Rural	47.91	< 0.001
	Urban	44.38	
**Educational attainment**	No Education	48.89	< 0.001
	less than 5 years	48.38	
	5–9 years	43.31	
	10 or more years	42.94	
**Marital Status**	Currently in marital union	45.61	< 0.001
	Not in marital union	50.65	
**Living alone**	No	46.58	< 0.001
	Yes	55.91	
**Currently working**	Yes	46.1	< 0.001
	No	47.74	
**Religion**	Hindu	46.84	< 0.001
	Muslim	45.37	
	Others	50.16	
**MPCE Quintile**	Poor	48.38	< 0.001
	Middle	46.26	
	Rich	45.44	
**ADL disability**	No	46.33	< 0.001
	Yes	50.07	
**IADL disability**	No	47.06	0.741
	Yes	46.75	
**SRH**	Good	40.76	< 0.001
	Poor	50.65	
**Current smoker**	No	47.13	0.703
	Yes	45.7	
**Alcohol use**	No	46.68	0.422
	Yes	46.95	
**Physical activity**	Frequent	44.24	< 0.001
	Ever	48.46	
	Never	51.5	
**Nutritional Status**	Normal weight	46.41	< 0.001
	Underweight	51.14	
	Overweight/obese	43.03	
**Regional geography**	Central	49.63	< 0.001
	North	62.14	
	East	54.28	
	North East	50.89	
	West	61.55	
	South	47.63	
**Total**		46.94	

*%* Percentage, *MPCE* Monthly per capita expenditure, *ADL* Activities of daily living, *IADL* Instrumental activities of daily living, *SRH* Self-Rated Health

Table 6 shows the logistic regression estimates for food insecurity by multimorbidity and other variables included in the study. The unadjusted Model 1 produced a significant association between multimorbidity and food insecurity. It was found that people with multimorbidity were 22% more likely to be food insecure than people without multimorbidity [uOR: 1.22; CI: 1.04–1.45]. Model 2 was adjusted for sociodemographic variables and the result remained the same, with a 6% increase in the odds of food insecurity [aOR: 1.28; CI: 1.09–1.50]. In Model 3, health related factors along with sociodemographic factors were adjusted and a similar significant association between multimorbidity and food insecurity was found [aOR: 1.23; CI: 1.05–1.43].

**Table 6:** Multivariable logistic regression estimates of the association between multimorbidity and food insecurity among socially disadvantaged people, 2017–18

Variables	Category	Model 1	Model 2	Model 3	Model 4	Model 5
		**uOR**	**aOR (95% CI)**	**aOR (95% CI)**	**aOR (95% CI)**	**aOR (95% CI)**
**Multimorbidity**	No	Ref	Ref			
	Yes	1.22*** (1.04,1.45)	1.28** (1.09,1.5)	1.23** (1.05,1.43)		
**Age (in years)**	45–59		Ref	Ref		Ref
	60 +		0.96 (0.87,1.01)	0.88** (0.80,0.97)		0.88** (0.80,0.97)
**Gender**	Female		Ref	Ref		
	Male		1.03 (0.92,1.16)	1.02 (0.92,1.14)	1.02 (0.92,1.14)	
**Residence**	Rural		Ref	Ref	Ref	
	Urban		0.85** (0.76,0.95)	0.92 (0.82,1.02)	0.92 (0.82,1.02)	0.92 (0.82,1.02)
**Education**	No Education		Ref	Ref	Ref	Ref
	less than 5 years		1.02 (0.9,1.14)	0.97 (0.87,1.09)	0.97 (0.87,1.09)	0.97 (0.87,1.09)
	5–9 years		0.83** (0.73,0.94)	0.83** (0.73,0.94)	0.83** (0.73,0.94)	0.83** (0.73,0.94)
	10 or more years		0.82* (0.65,1.03)	0.76* (0.62,0.95)	0.76* (0.62,0.95)	0.76* (0.62,0.94)
**Marital Status**	Currently in union		Ref	Ref	Ref	Ref
	Not in union		1.13** (1.01,1.28)	1.08 (0.96,1.22)	1.08 (0.96,1.22)	1.09 (0.96,1.22)
**Living alone**	No		Ref	Ref	Ref	Ref
	Yes		1.21 (1,1.47)	1.27** (1.04,1.56)	1.27** (1.04,1.55)	1.27** (1.04,1.55)
**Currently working**	Yes		Ref	Ref	Ref	Ref
	No		1.03 (0.93,1.14)	0.98 (0.9,1.08)	0.98 (0.9,1.08)	0.98 (0.9,1.08)
**Religion**	Hindu		Ref	Ref	Ref	Ref
	Muslim		0.93 (0.74,1.16)	0.92 (0.79,1.08)	0.92 (0.78,1.08)	0.92 (0.78,1.08)
	Others		1.23* (1.01,1.51)	1.25* (1.03,1.52)	1.25* (1.03,1.52)	1.25* (1.03,1.51)
**MPCE Quintile**	Rich		Ref	Ref	Ref	Ref
	Poor		1.16 *** (1.05,1.28)	1.11* (1,1.23)	1.11* (1,1.23)	1.11* (1,1.23)
	Middle		1.06 (0.92,1.21)	1.02 (0.88,1.17)	1.02 (0.88,1.17)	1.01 (0.88,1.17)
**ADL disability**	No			Ref	Ref	Ref
	Yes			1.2*** (1.06,1.37)	1.21** (1.06,1.37)	1.2** (1.06,1.37)
**IADL disability**	No			Ref	Ref	Ref
	Yes			0.8*** (0.72,0.88)	0.8*** (0.72,0.88)	0.8*** (0.72,0.88)
**SRH**	Good			Ref	Ref	Ref
	Poor			1.36*** (1.25,1.49)	1.36*** (1.25,1.49)	1.36*** (1.24,1.49)
**Current smoker**	No			Ref	Ref	Ref
	Yes			1.06 (0.96,1.18)	1.06 (0.96,1.18)	1.06 (0.95,1.18)
**Alcohol use**	No			Ref	Ref	Ref
	Yes			1.1* (0.99,1.23)	1.1* (0.99,1.23)	1.1* (0.98,1.23)
**Physical activity**	Frequent			Ref	Ref	Ref
	Ever			1.25*** (1.12,1.39)	1.25 (1.12,1.39)	1.25*** (1.12,1.39)
	Never			1.35*** (1.23,1.49)	1.35 (1.23,1.49)	1.35*** (1.23,1.49)
**Nutritional Status**	Normal weight			Ref	Ref	Ref
	Underweight			1.18** (1.07,1.3)	1.18** (1.07,1.3)	1.18** (1.07,1.3)
	Overweight/obese			0.86* (0.76,0.98)	0.86* (0.76,0.98)	0.87* (0.77,0.98)
**Regional geography**	Central		Ref	Ref	Ref	Ref
	North		0.59*** (0.53,0.66)	0.6*** (0.54,0.67)	0.6*** (0.54,0.67)	0.6*** (0.54,0.67)
	East		0.8* (0.73,0.89)	0.84** (0.76,0.93)	0.84** (0.76,0.93)	0.84** (0.76,0.93)
	Northeast		0.93*** (0.82,1.05)	1.02 (0.9,1.15)	1.02 (0.9,1.15)	1.01 (0.89,1.15)
	West		0.62** (0.55,0.7)	0.64*** (0.56,0.72)	0.64 ***(0.56,0.72)	0.63*** (0.56,0.72)
	South		1.12*** (1,1.26)	1.2** (1.06,1.35)	1.2 **(1.06,1.35)	1.19** (1.06,1.34)
**Multimorbidity # Age group**	Yes # Aged 45–59 years				Ref	
	No # Aged 45–59 years				0.77* (0.59,0.99)	
	No # Aged over 60 years				0.69* (0.54,0.89)	
	Yes # Aged over 60 years				0.83** (0.71,0.98)	
**Multimorbidity # Gender**	Yes # Women					Ref
	No # Women					0.88 (0.72,1.08)
	No # Men					0.87 (0.71,1.05)
	Yes # Men					1.21** (1.02,1.43)
Pseudo R^2^		0.0011	0.0153	0.262	0.263	0.264

Model 1: Unadjusted model

Model 2: Adjusted for individual along with household factors (age, gender, place of residence, education, marital status, working status, wealth quintile, religion, living arrangements, region)

Model 3: Adjusted for model 2 and health and behavioral factors (functional disability of ADL & IADL, SRH, smoking and alcohol use, physical activity, and nutritional status)

Model 4: Adjusted model showing interaction of multimorbidity and age group

Model 5: Adjusted model showing interaction of multimorbidity and gender

*%* Percentage, *aOR* adjusted Odds Ratio, *CI* Confidence interval, *MPCE* Monthly per capita expenditure, *ADL* Activities of daily living, *IADL* Instrumental activities of daily living, *SRH* Self-Rated Health

* *p* < 0.05; **p < 0.005; ***p < 0.001

Model 4 shows the interactive effect of multimorbidity with age group on food insecurity. People aged 60 years or more with multimorbidity were 17% significantly lower likelihood to be food insecure than people aged 45–59 years with multimorbidity [aOR: 0.83; CI: 0.71–0.94]. Model 5 illustrates the interactive effect of multimorbidity with the gender of older people on food insecurity. Men having multimorbidity were 1.21 times significantly more likely to be food insecure than women with multimorbidity [aOR: 1.21; CI: 1.02–1.43].

## Discussion

This study examines the association between multimorbidity and food insecurity of socially disadvantaged middle and old-aged adults in India. Using large-scale survey data, we document significant and noteworthy detriments in food security among those socially disadvantaged individuals having multimorbidity. The association was statistically significant independent of socioeconomic and health measures, suggesting the role of multimorbidity in determining food insecurity among the older population. While looking at the gender and age aspect, our study found that men and middle-aged individuals with multimorbidity had a higher prevalence of food insecurity. At the same time, logistic regression analysis showed that multimorbidity among men and middle-aged is significantly associated with food insecurity among socially disadvantaged groups.

Our findings on the multimorbidity and association with food insecurity is also supported by the existing studies. Jih and team (2018) suggested that multimorbidity condition can directly impacts the household budgets irrespective of age which can further increase the risk of food insecurity [13]. This can also be true for the socially disadvantageous group as these peoples lacks basic needs *i.e.,* food, education and decent livelihood. Saying so, burden of chronic morbidities can further put excess pressure on household budged possibly increasing the food insecurity [33, 36].

While our finding on gender aspect suggested higher food insecurity prevalence among men individuals with multimorbidity than women was consistent with existing literate [44–46]. A study reported that men with disability and physical health problems were more likely to be undernourished and face food insecurity [45]. Although, in India, where the patriarchal social attributes are prominent, gender discrimination is a recognized situation which is also reflected through more economic dependence, higher morbidity prevalence, and experiencing food insecurity among all ages of women [47–49]. Thus, women with chronic health problems, particularly those belonging to disadvantaged groups, may experience higher food insecurity [31, 50, 51]. However, our findings suggest that males with multimorbidity experience more heightened food insecurity than women. Although our results contradict previous studies, our study focused specifically on the underprivileged group, which may explain some of the contradiction. As previously stated, men members of disadvantaged groups may experience more economic constraints and health-related concerns than women members of disadvantaged groups. While, in India, there are numerous programs and policies aimed at women and the elderly addressing nutrition, pensions, and health-related issues [52], the men members of these socially disadvantaged groups may go unnoticed, leaving socially disadvantageous leadings to males more vulnerable to poor health and food insecurity.

While focusing on the age groups, our results on a higher prevalence of food insecurity among middle-aged adults with multimorbidity than the older adult were also in line with previous studies [13, 53, 54]. The results contribute to previous research by identifying that the disability and health issues may pose the greatest vulnerability of food insecurity among the structurally disadvantaged group, especially in the working middle-age range [45]. From a comparative perspective, food insecurity among middle-aged adults is similar in magnitude to deprived racial and ethnic groups in the western world [45, 54–58]. Although the mechanism of food insecurity in middle-aged population remains obscure in comparison to earlier and later ages, there are factors in the literature that may explain the ambiguity. It is evident that the age range of 45 to 59 years represents an individual's transition period, during which the individual experiences the end of his or her early adulthood and in the process of entering old age. Middle-aged individuals undergo physiological and psychological changes throughout this period and experience social and employment-related changes that have a direct or indirect effect on their health and food security [59, 60]. The onset of chronic illness and functional limits, including reduced mobility and mass strength, occurs during this middle-aged period, which is associated with food insecurity. Research conducted in the United States of America concluded that midlife changes are likely to increase the effectiveness of health problems, hence raising the probability of food insecurity [54]. Another potential explanation for the association between food insecurity and multimorbidity in middle-aged adults found in the existing research is social roles, including financial instability, parenthood, and other types of caring [61]. Middle-aged adults care for their children and elderly parents concurrently, resulting in increased financial stress and responsibility. These economic concerns and commitments have been linked to poor physical and psychological health, further increasing the likelihood of food insecurity and poor diet quality in middle-aged people [62, 63]. While in midlife, the emergence of physical health problems increases the risk of lost work time and a lower likelihood of re-employment, which may predispose middle-aged persons to food insecurity [64]. Caring for younger children and the elderly is a social obligation in the Indian system [65]. While socially disadvantaged middle-aged persons may experience a variety of economic difficulties, these burdens may increase their health issues, making them even more vulnerable to food insecurity [66].

## Strength and limitations

Our study has several strengths. Firstly, this study attempted to fill the gap in literature on association between food insecurity and multimorbidity among socially disadvantaged people in India. Secondly, the use of recently released nationally representative cross-sectional dataset allow us to obtain robust estimates of the variables under consideration. However, this study also met with some limitations. The cross-sectional nature of data does not infer any causal relationship; further longitudinal data can give us more insight in investigating the causal relationship between food insecurity and multimorbidity. The information on multimorbidity was based on nine self-reported chronic conditions resulting in misclassification bias. Similarly, recall bias might affect the quality of data on self-reported health activities such as physical activity, ADL, IADL etc.

## Conclusions

The outcomes of this research indicate a link between multimorbidity and food insecurity among India's structurally disadvantaged adults. The socially disadvantage groups as more likely to experience the multiple chronic morbidities, that may further affects their quality of diet and, nutritionally inadequate meals to maintain caloric intake, placing them at risk for a range of detrimental health consequences. Additionally, food insecurity among middle-aged adults and males with multimorbidity in disadvantage groups might provide a new dimension, emphasizing the need of considering social background as an important identifier for healthcare system. As a result, enhancing health care system may help to reduce food insecurity in individuals who are multimorbid.

## Data Availability

The data that support the findings of the study is publicly available from the international institute for population sciences website: https://www.iipsindia.ac.in/lasi.
